# Sociodemographic and Clinical Profile of Perpetrators of conjugal Homicides

**DOI:** 10.1192/j.eurpsy.2024.362

**Published:** 2024-08-27

**Authors:** W. Haouari, S. Omri, A. Labyadh, R. Feki, I. Gassara, L. Zouari, J. Ben Thabet, N. Charfi, M. B. Maalej, N. Smaoui, M. Maalej

**Affiliations:** Psychiatrie C, Hedi Chaker university Hospital, Sfax, Tunisia

## Abstract

**Introduction:**

Conjugal homicide refers to the act of killing a current or former intimate life partner, regardless of their marital status. This type of behavior is still inadequately addressed by prevention programs, as it is often regarded as exceptional compared to other forms of domestic violence.

**Objectives:**

To describe the sociodemographic, clinical, and criminological data of individuals who commit spousal homicide.

**Methods:**

This is a retrospective descriptive study of 21 psychiatric expertise files conducted between January 2002 and September 2023 in the psychiatric department of Hedi Chaker Hospital in Sfax. Sociodemographic, clinical, and criminological data were collected from criminal psychiatric expertise files and supplemented with information from medical records.

**Results:**

The perpetrators of spousal homicide were predominantly male (85.7%), had an educational level above secondary school (57.1%), were married (85.7%), and had an average age at the time of the act of 40.3 years. Most of them had no psychiatric history (81%) or legal history(85%), and only 19% were using psychoactive substances.

Among the perpetrators, 66.7% had a history of violence against their partners, with threats of homicide in 19% of cases. The majority of homicides occurred during the day (42.9%), in a public place (28.6%), were perpetrated in isolation (95.2%), happened impulsively (47.6%), and employed a single method (81%), with knives being the primary weapon (42.9%). In 76.2% of cases, the perpetrators of spousal homicides were found criminally responsible, while 19% were hospitalized in a psychiatric setting as part of a judicial non-prosecution decision.

**Conclusions:**

The study of specific characteristics of spousal homicides holds crucial importance for the early detection of domestic violence situations that carry a lethal risk. By highlighting these particularities, it enables the development of more targeted prevention strategies.

**Disclosure of Interest:**

None Declared

